# Association of Blood Cadmium Level with Cardiometabolic Risk Factors and Liver Enzymes in a Nationally Representative Sample of Adolescents: The CASPIAN-III Study

**DOI:** 10.1155/2013/142856

**Published:** 2013-05-16

**Authors:** Roya Kelishadi, Ahmadreza Askarieh, Mohammaad Esmaeil Motlagh, Mohammadhasan Tajadini, Ramin Heshmat, Gelayol Ardalan, Sepideh Fallahi, Parinaz Poursafa

**Affiliations:** ^1^Child Growth and Development Research Center, Isfahan University of Medical Sciences, Isfahan, Iran; ^2^Bureau of Population, Family and School Health, Ministry of Health and Medical Education, Tehran, Iran; ^3^Ahvaz Jundishapur University of Medical Sciences, Ahvaz, Iran; ^4^School of Pharmacy and Isfahan Pharmaceutical Sciences Research Center, Isfahan University of Medical Sciences, Isfahan, Iran; ^5^Chronic Diseases Research Center, Endocrinology and Metabolism Population Sciences Institute, Endocrinology and Metabolism Research Institute, Tehran University of Medical Sciences, Tehran, Iran; ^6^Environment Research Center, Isfahan University of Medical Sciences, Isfahan 81676-36954, Iran

## Abstract

*Introduction*. This study aimed to determine the association of blood cadmium level with cardiometabolic risk factors and liver enzymes in adolescents. *Methods*. This case control study comprised 320 Iranian adolescents, 160 with metabolic syndrome and an equal number of controls. They were selected from participants of a nationwide survey entitled the CASPIAN-III study. Cadmium was measured by atomic absorption method. *Results*. The mean age of the case and control groups was not significantly different (15.3 ± 2.6 versus 14.63 ± 2.5 years, resp., *P* > 0.05). The mean cadmium level was near double-fold higher than the standards of the World Health Organization, without significant difference between the MetS and control groups (10.09 ± 2.21, 9.97 ± 2.38 **μ**g/L, resp., *P* > 0.05). Cadmium level had positive but nonsignificant correlations with diastolic blood pressure, serum triglycerides, fasting blood glucose, LDL-C, and liver enzymes. *Conclusion*. Cadmium level had positive but nonsignificant association with some cardiometabolic risk factors and liver enzymes. The associations did not reach statistical significant level, and this may be because of the high levels of cadmium in both groups studied or because of the young age group of participants. Controlling environmental pollutants shall be a priority for the prevention of chronic diseases.

## 1. Introduction

Metabolic syndrome (MetS) is an emerging health problem at global level and increases the risk of most chronic diseases. It origins from early life and consists of various components including obesity, elevated blood pressure, elevated serum glucose, and dyslipidemia in terms of increased triglycerides and reduced high-density lipoprotein cholesterol (HDL cholesterol) levels [1]. It is no more limited to the western countries and adult populations [2, 3]. Asians have an ethnic predisposition to MetS, and it is one of health concerns in Iran [4, 5]. 

MetS is a multifactorial condition, and in addition to genetic and lifestyle factors, environment influences the development of this disorder [6]. Heavy metals are one of the environmental factors that may have a role in this regard.

Heavy metals or toxic metals such as mercury, lead, and cadmium have no biological function in human system and are potentially toxic even at trace concentrations. Cadmium can enter into blood stream by eating and drinking cadmium-contaminated food or water and/or by breathing cadmium-contaminated air [7–9]. Lee and Kim reported for the first time that blood cadmium level is a risk factor for MetS [9]. Various studies showed that urinary cadmium levels are significantly and dose dependently associated with both impaired fasting glucose and diabetes and even can lead to diabetic nephropathy [10, 11]. A study in Pakistan revealed that high cadmium levels in biological samples of diabetic women may play a role in the pathogenesis of diabetes mellitus and may also impact on their neonates [12]. With the advent of large-scale metal mining and smelting, as well as fossil fuel combustion in the industrial countries, the emission rate of heavy metals has increased dramatically [13].

Both MetS and cadmium exposure and accumulation in the body start at young age [14, 15]. Therefore, a relationship may exist between cadmium and MetS from childhood.

This study aimed to compare the serum cadmium level, cardiometabolic risk factors, and liver function tests in adolescents with and without MetS.

## 2. Methods

This case control study was conducted as a substudy of the third survey of the national school-based surveillance system entitled Childhood and Adolescence Surveillance and PreventIon of Adult Noncommunicable disease (CASPIAN-III) (Caspian is the name of the world's largest lake, located in Northern Iran) study. The main study was approved by the institutional review boards at national and provincial levels. Written consent and oral assent were obtained from students and their parents, respectively. The current substudy was conducted on blood samples collected in the main study and was approved by the Ethics Committee of Isfahan University of Medical Sciences. This study was performed in accordance with the ethical standards of the Helsinki Declaration.

The main study was conducted as a school-based nationwide health survey among 5570 students aged 10–18 years, who were recruited by multistage random cluster sampling from urban and rural areas of 27 provincial counties in Iran. Those students with history of any acute or chronic diseases and any medication use were not included in the study [16].

A trained team of health professionals conducted the physical examination under standard protocols by using calibrated instruments. Weight, height, and waist circumference (WC) were measured. Body mass index (BMI) was calculated as weight (Kg) divided by height squared (m^2^). Blood pressure was measured under standard protocol [17]. 

For blood sampling, students were invited to the nearest health center to the school. Fasting venous blood samples were centrifuged, and fresh sera were analyzed for fasting blood glucose (FBG), lipid profile, and liver function tests, that is, alanine aminotransaminase (ALT) and aspartate aminotransaminase (AST) by using Pars Azmoon reagent kits (Tehran, Iran). For measuring cadmium, frozen sera of 160 participants with MetS and an equal number of healthy controls were used. Cadmium levels were determined by atomic absorption spectrophotometer by using hollow cathode lamps. 

Similar to the first survey of CASPIAN study [18], we used the definition provided by Cook et al. [19]. This definition is based on criteria analogous to that of the National Cholesterol Education Program Expert Panel on Detection, Evaluation, and Treatment of High Blood Cholesterol in Adults Adult Treatment Panel III (ATP III) [20]. It defines the MetS as having at least three of the following criteria: WC was at or above the 90th percentile value for age and sex; SBP and DBP were at or above the 90th percentile for age, sex, and height; the midpoint value for HDL-C (≤40 mg/dL) was used as a 10th percentile value; the midpoint value for TG (≥110 mg/dL) was taken as the 90th percentile value for age. FBG levels of ≥100 mg/dL were considered to be high [21].

### 2.1. Statistical Analyses

Statistical analyses were performed using SPSS statistical package version 18 for Windows. Chi-square and independent sample *t*-tests were used to compare categorical and quantitative data, respectively. Correlation models were used to assess the relationships between the diagnostic components of MetS and cadmium concentration. *P* values of <0.05 were considered as statistically significant.

## 3. Results

The study population consisted of 320 adolescents (160 with MetS and 160 healthy controls). The mean age of the case and control groups was not significantly different (15.3 ± 2.6 versus 14.96 ± 2.51 years, resp., *P* > 0.05). The mean cadmium level was near double fold higher than the standards of the World Health Organization [22], without significant difference between the MetS and control groups (10.09 ± 2.21, 9.97 ± 2.38 *μ*g/L, resp., *P* > 0.05).

Table 1 presents the characteristics of the study population. BMI, total cholesterol (TC), TG, FBG, ALT, SBP, and DBP were significantly higher in the MetS group than in controls. The corresponding figure was not significantly different for AST, HDL-C, and low density lipoprotein cholesterol (LDL-C).

According to the regression analysis, cadmium level had positive but nonsignificant relationship with LDL-C, TG, FBG, ALT, AST, and DBP (Table 2).

## 4. Discussion

We investigated the association of cadmium level with cardiometabolic risk factors, MetS, and liver function tests in a nationally representative sample of Iranian adolescents. To the best of our knowledge, this study is the first of its kind in the pediatric age group. Cadmium level was near twofold higher than standard levels [22] in all of the population studied. However, cadmium level was not significantly different among adolescents with and without MetS. Likewise, cadmium had positive, but nonsignificant association with liver function tests and most cardiometabolic risk factors. This nonsignificant association may be because of high levels of cadmium in both groups with and without MetS. In addition, it is suggested that the adverse health effects of cadmium on cardiometabolic risk factors would develop over time and may be nonsignificant in adolescence.

Children can be exposed to cadmium through contaminated air, water, soil, food, consumer products, and second-hand smoke [23]. The estimated half-life of cadmium is about 10 to 30 years, and over time, it accumulates in different organs as kidney, liver, bone marrow, and muscles, and these organs could be a source of cadmium continuously released into the bloodstream [24, 25]. 

Contrary to our results, a study in Korea revealed that blood cadmium levels increased the risk of MetS in adults [9]. It is well documented that chronic cadmium exposure may cause impaired fasting glucose and diabetes in humans [26, 27]. Heme oxygenase-2 (HO-2) acts as a protective factor against type-2 diabetes and obesity; cadmium has the propensity to alter its catabolism and may increase the risk of diabetes [28]. We did not find any significant association of cadmium with MetS and FBG; this may be because of the young age group studied; such association may develop over time.

Some studies have reported blood cadmium level as a risk factor for prehypertension in both women and men [29]. Cadmium concentrates in the kidney and may induce proteinuria and renal dysfunction; in turn it may cause hypertension. Moreover, renal cadmium reduces CYP4A11 and PPARs, which may be related to hypertension and sodium retention [30, 31]. We found positive association between cadmium and blood pressure, but the weak and nonsignificant correlation may be because of the young age of the study participants, and longitudinal studies are necessary to assess the long-term effects of cadmium on blood pressure.

In our study, the association of cadmium level with serum lipid profile was weakly positive, but nonsignificant; this may be because in both groups with and without MetS, cadmium level was considerably high without significant difference between the two groups. Experimental studies have shown that cadmium exposure induces alterations in lipid profiles [32–34]. No epidemiological study has been performed in this regard. However, some studies showed that cadmium levels in blood and urine are independent factors associated with the development of atherosclerotic plaques by the influence on selected lipid metabolism parameters [35–37].

Environmental factors have various health impacts on risk factors of noncommunicable diseases [38] even in children and adolescents [39, 40]. Different sources of pollutants should be controlled to prevent their short-term and long-term adverse health effects. 

### 4.1. Study Limitations and Strengths

The main limitation of this study is its cross-sectional nature, so the associations of different variables should be considered with caution. The study strengths are the novelty of studying the association of cadmium with cardiometabolic risk factors and liver enzymes in the pediatric age group and using data of a nationally representative group of adolescents, which would increase the generalizability of the study findings.

## 5. Conclusion

Cadmium level was considerably high in both groups of adolescents with and without MetS. It had positive but nonsignificant association with cardiometabolic risk factors and liver enzymes. This finding may be because of the high levels of cadmium in both groups studied or because of the young age group of participants. Controlling environmental pollutants shall be considered as a health priority for primordial/primary prevention of noncommunicable diseases.

## Figures and Tables

**Table 1:** Characteristics of adolescents with and without metabolic syndrome: the CASPIAN-III Study.

	Metabolic syndrome group	Control group	*P* value
Age (years)	15.3 ± 2.6	14.96 ± 2.51	0.13
Cadmium (*μ*g/L)	9.97 ± 2.38	10.09 ± 2.21	0.65
Systolic blood pressure (mm Hg)	122 ± 11.03	101.64 ± 14.57	<0.0001
Diastolic blood pressure (mm Hg)	79.32 ± 6.85	64.52 ± 11.08	<0.0001
Total Cholesterol (mg/dL)	162.9 ± 40	147.45 ± 24.26	<0.0001
HDL-C (mg/dL)	41.56 ± 15.65	41.45 ± 9.82	0.939
LDL-C (mg/dL)	91.56 ± 24.14	90.65 ± 20.31	0.71
Triglycerides (mg/dL)	130.9 ± 67	79.81 ± 20.42	<0.0001
Fasting blood glucose (mg/dL)	97.4 ± 16.95	81.3 ± 6.3	<0.0001
Alanine aminotransaminase (U/L)	23.38 ± 5.17	16.84 ± 5.88	<0.0001
Aspartate aminotransaminase (U/L)	24.9 ± 5.11	23.31 ± 5.24	0.2

**Table 2:** Linear regression analysis of cadmium with cardiometabolic risk factors and liver enzymes: the CASPIAN-III Study.

	Cadmium level	
	Beta	*P* value
Body mass index	0.006	0.92
Systolic blood pressure	−0.05	0.52
Diastolic blood pressure	0.10	0.24
Total Cholesterol	0.65	0.47
HDL-C	−0.03	0.56
LDL-C	0.04	0.62
Triglycerides	0.02	0.78
Fasting blood glucose	0.042	0.58
Aspartate aminotransaminase	0.06	0.92
Alanine aminotransaminase	0.08	0.82
